# Excessive Pregestational Weight and Maternal Obstetric Complications: The Role of Adipokines

**DOI:** 10.3390/ijms241914678

**Published:** 2023-09-28

**Authors:** Jorge Valencia-Ortega, Juan Mario Solis-Paredes, Renata Saucedo, Guadalupe Estrada-Gutierrez, Ignacio Camacho-Arroyo

**Affiliations:** 1 Unidad de Investigación en Reproducción Humana, Instituto Nacional de Perinatología-Facultad de Química, Universidad Nacional Autónoma de México, Mexico City 11000, Mexico; j.valencia.o@hotmail.com; 2 Department of Reproductive and Perinatal Health Research, Instituto Nacional de Perinatología Isidro Espinosa de los Reyes, Mexico City 11000, Mexico; juan.solis@inper.gob.mx; 3 Unidad de Investigación Médica en Enfermedades Endocrinas, Hospital de Especialidades, Centro Médico Nacional Siglo XXI, Instituto Mexicano del Seguro Social, Mexico City 06720, Mexico; sgrenata@yahoo.com; 4 Research Division, Instituto Nacional de Perinatología, Mexico City 11000, Mexico; gpestrad@gmail.com

**Keywords:** maternal obesity, pregnancy complications, adipokines, cytokines

## Abstract

There is a high frequency of overweight and obesity in women of reproductive age. Women who start pregnancy with overweight or obesity have an increased risk of developing maternal obstetric complications such as gestational hypertension, pre-eclampsia, gestational diabetes mellitus, postpartum hemorrhage, and requiring C-section to resolve the pregnancy with a higher risk of C-section surgical site infection. Excessive weight in pregnancy is characterized by dysregulation of adipokines, the functions of which partly explain the predisposition of pregnant women with overweight or obesity to these maternal obstetric complications. This review compiles, organizes, and analyzes the most recent studies on adipokines in pregnant women with excess weight and the potential pathophysiological mechanisms favoring the development of maternal pregnancy complications.

## 1. Introduction

Excess body weight results from the overaccumulation of fat caused by an energy imbalance between intake and expenditure of calories [1]. According to the World Health Organization, excessive weight is classified based on body mass index (BMI) as overweight or pre-obesity (25.0–29.9 kg/m^2^), obesity class I (30.0–34.9 kg/m^2^), obesity class II (35.0–39.9 kg/m^2^), and obesity class III (≥40.0 kg/m^2^) [2]. This metabolic condition represents a priority public health issue as it considerably increases the risk of cardiovascular disease, diabetes mellitus, and certain types of cancer [1,3].

**Figure 1:**
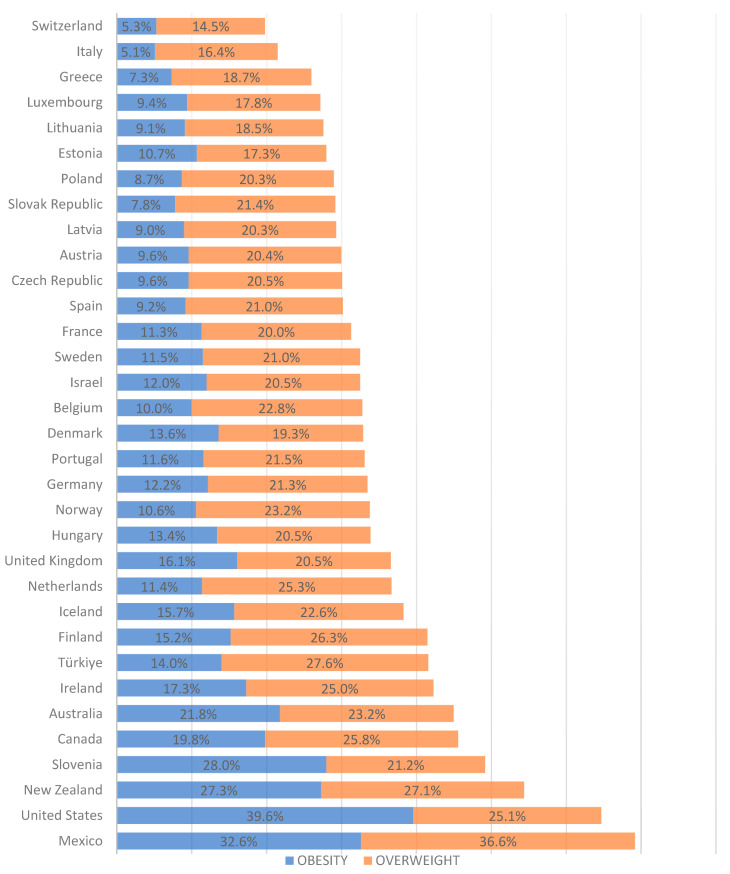
Prevalence of excessive weight in women. Prevalence data are shown only for member countries of the Organization for Economic Cooperation and Development. Women aged 18 to 44 were included in most countries (Supplementary Table S1). Data are from the Global Obesity Observatory [5].

The prevalence of overweight and obesity has substantially augmented in all countries, although with more accelerated increases in certain regions, particularly in East and South Asia [4]. This increase is reflected in the prevalence of overweight and obesity in women, which exceeds 60% in countries such as the USA and Mexico (Figure 1) [5]. This increase mainly occurs in women of reproductive age, which is alarming because excessive pregestational weight is associated with the development of maternal pregnancy-related complications and, according to the Development Origin of Health and Diseases theory, has significant consequences on the short- and long-term health of offspring [6,7].

Although the mechanisms underlying the association between excessive pregestational weight and the risk of maternal obstetric complications have not been fully elucidated, it is known that overweight and obesity are characterized by dysregulation in the production of various adipokines that could play an essential role in this association. In this sense, this review organizes and analyzes the most recent studies on adipokines in pregnant women with excess weight and the potential mechanisms favoring the development of maternal pregnancy complications.

## 2. Search Strategies

Individually, authors retrieved articles from Pubmed using the following search terms: “adipose tissue”, “maternal obesity”, “maternal overweight”, “adipokines”, “gestational diabetes mellitus”, “postpartum hemorrhage”, “C-section”, “cesarean section”, “C-section surgical site infection”, “preeclampsia”, “endothelial dysfunction”, “glucose metabolism”, “insulin resistance”, “myometrial contractility”, “uterine myometrial contractions”, and “failed labor induction”. Only relevant English-language articles were chosen. It is important to note that in order to compile the most recent studies and count a considerable number of papers in each section of the review, only those published between 2017 and 2023 that evaluated the risk of maternal obstetric complications in excess-weight women were included, and for the section on adipokine concentrations in pregnant women with excessive weight, works published between 2013 and 2023 were included since there are few studies on the subject. For the section on pathophysiological mechanisms of adipokines in maternal obstetric complications, no publication year limit was applied to give credit to pioneering work and avoid secondary citations.

## 3. Risk of Maternal Obstetric Complications in Pregnant Women with Excess Weight

**Figure 2:**
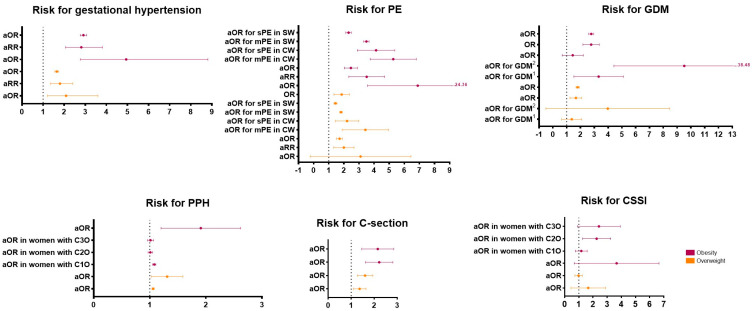
Risk of maternal obstetric complications in pregestational excess-weight women. All odds ratios (ORs) and relative risks (RRs) were compared to normal-weight pregnant women [8,9,10,11,12,13,14,15,16,17,18,19,20,21]. a: adjusted; PE: pre-eclampsia; mPE: mild pre-eclampsia; sPE: severe pre-eclampsia; SW: Swedish women; CW: Chinese women; GDM: gestational diabetes mellitus; ^1^: GDM that required only dietary modifications; ^2^: GDM that required insulin therapy; PPH: postpartum hemorrhage; C1O: class 1 obesity; C2O: class 2 obesity; C3O: class 3 obesity; and CSSI: C-section surgical site infection.

Gestational hypertension, pre-eclampsia (PE), gestational diabetes mellitus (GDM), postpartum hemorrhage (PPH), the requirement of a C-section to resolve the pregnancy, and C-section surgical site infection (CSSI) are among the most common maternal obstetric complications. Figure 2 shows the risk for these complications in women with overweight or obesity. It can be seen that both conditions increase the risk of gestational hypertension, PE, GDM, and requiring a C-section to terminate the pregnancy. However, the risk is higher in women with obesity. Excessive weight does not considerably increase the risk of PPH and CSSI. Endocrine changes in adipose tissue partly explain the role of excessive weight in predisposing to these complications.

## 4. Adipose Tissue, Adipokines, and Excessive Weight in Pregnancy

Adipose tissue is composed of adipocytes and the stromal vascular fraction comprising blood and blood vessels, nervous tissue, and different cell types such as fibroblasts, macrophages, and other immune cells, which, through various mediators, support the differentiation of pre-adipocytes to adipocytes. Adipose tissue is not a single organ located in a specific anatomical space but is distributed throughout the body in various anatomical and functional depots. It is divided into three subtypes: white, brown, and beige. The latter two play important roles in thermogenesis and fat oxidation, respectively, while white adipose tissue is the main site of lipid storage and mobilization. This tissue buffers the toxic effects of excess circulating lipids by storing free fatty acids in the form of triglycerides and releasing them for use by peripheral tissues in periods of energy deprivation. Anatomically, white adipose tissue is classified into subcutaneous and visceral tissue. Subcutaneous adipose tissue is the largest reservoir of fatty acids, and its functions depend on its body location so that lower body subcutaneous adipose tissue is more sensitive to insulin and does not release fatty acids as readily, whereas upper body subcutaneous adipose tissue is relatively insulin-resistant and very prone to releasing fatty acids. Visceral adipose tissue is divided into epicardial, omental, mesenteric, perirenal, and gonadal and is associated with the development of obesity-related disorders, probably by the release of fatty acids directly into the portal circulation. Many characteristics of adipose-tissue deposits, such as location, size, and metabolic functions, are influenced by genetic background and sex [22,23]. It is now widely accepted that adipose tissue is a metabolically active organ that secretes more than 600 mediator peptides, collectively known as adipocytokines or adipokines, which play an important role in modulating maternal metabolism and subsequently influence fetal development and growth [24].

Early pregnancy is characterized by high insulin sensitivity that favors energy storage through increased body fat, which accounts for about one-third of total gestational weight gain. These fat stores represent the main source of energy for the mother and fetus in late pregnancy and lactation [22]. Physiologically, there is a shift in the redistribution of adipose tissue from early to late pregnancy, which is characterized by increased visceral fat accumulation and decreased subcutaneous fat accretion [25].

Body fat gain depends on the nutritional status before pregnancy, so women with normal weight tend to gain more adipose tissue than those with obesity, which is explained by the insulin resistance that characterizes this condition [26]. In normal-weight women, it is mainly the subcutaneous adipose tissue that increases its mass, while in women with obesity, this preferentially occurs in the visceral adipose tissue. In comparison to subcutaneous fat, visceral fat is metabolically more active. It has higher endocrine activity through the production of a large number of adipokines that affect glucose, protein, and lipid metabolism, which are implicated in the development of insulin resistance, dyslipidemia, inflammation, and other clinical features associated with increased cardiometabolic risk [22,27]. Leptin, adiponectin, resistin, tumor necrosis factor-alpha (TNF-α), interleukin 6 (IL-6), visfatin, and apelin are among the main adipokines produced by the adipose tissue. Table 1 shows the most recent articles on maternal concentrations of these adipokines according to nutritional status.

In summary, it is not clear whether overweight pregnant women have higher leptin concentrations. However, in the case of pregnant women with obesity, leptin concentrations are higher throughout pregnancy compared to normal-weight women. It is also unclear whether adiponectin concentrations differ between overweight and normal-weight pregnant women, although one longitudinal study found that adiponectin concentrations were lower throughout pregnancy in overweight women. Four of the six cross-sectional studies in obese pregnant women report no significant difference in adiponectin concentrations in the third trimester of gestation; however, three longitudinal studies observed lower concentrations of this adipokine throughout pregnancy. Four cross-sectional studies show that third-trimester resistin concentrations do not differ between women with overweight or obesity and normal-weight women. One cross-sectional and one longitudinal study indicate no differences in IL-6 concentrations between these groups. The studies of TNF-α, visfatin, and apelin are very few, so it is not possible to highlight any trend.

It is necessary to mention that a previous review reported findings similar to those of the present work: higher leptin concentrations, lower adiponectin, and conflicting results for resistin, TNF-α, and IL-6 in pregnant women with obesity compared to women with normal weight [42]; however, the present article proposes the pathophysiological mechanisms of dysregulated adipokines leading to obstetric complications. Condensing this evidence, we can state that pregestational obesity is associated with higher leptin and lower adiponectin concentrations during pregnancy and that more detailed studies are needed to clarify whether the concentrations of other adipokines are altered in maternal obesity. Therefore, the following section only focuses on the pathophysiological mechanisms leptin and adiponectin may trigger in maternal obstetric complications.

## 5. Potential Pathophysiological Mechanisms of Leptin and Adiponectin in Maternal Obstetric Complications

### 5.1. Leptin

Leptin is a 16 kDa protein hormone produced by white adipose tissue, placenta, and other tissues [43,44]. The main function of leptin is the regulation of body weight and metabolism at the central level, although it also modulates immune response and has reproductive functions at the level of implantation and embryonic development [45,46,47]. In humans, leptin acts in the brain to reduce food intake and increase energy expenditure [48,49]. This central regulation of leptin energy occurs at the level of the preoptic area, arcuate nucleus, and other hypothalamic regions [50]. Excessive weight is associated with a chronic inflammatory response characterized by altered concentrations of inflammatory markers such as TNF-α, IL-6, IL-18, and C-reactive protein, and activation of pro-inflammatory signaling pathways at the central nervous system level involving the suppressor of cytokine signaling 3 (SOCS3) [51,52,53]. 

Specifically, up-regulation of SOCS3 in hypothalamic regions may result in altered leptin signaling known as leptin resistance, which induces an exacerbated leptin production (hyperleptinemia) [54] and, in turn, amplifies the negative effects of this adipokine (described later) and the chronic pro-inflammatory state because leptin activates macrophages, dendritic cells, natural killer cells, and T cells [47]. Moreover, leptin resistance in the hypothalamus alters the central regulation of appetite and probably favors hyperphagia and excessive gestational weight gain [55,56], resulting in a vicious pro-inflammatory circle [57,58]. It is worth mentioning that, during pregnancy, a progressive physiological increase in maternal leptin concentrations is observed, and it is considered responsible for the energy changes associated with pregnancy [59,60].

Regarding the role of leptin in gestational hypertension and PE, it has been observed that, in a murine model, leptin infusion in mid-pregnancy induces endothelial dysfunction and hypertension. Interestingly, this does not occur in knockout mice for the endothelial mineralocorticoid receptor [61], which is consistent with studies in nonpregnant mice in which leptin infusion induced endothelial dysfunction via leptin-mediated aldosterone production and endothelial mineralocorticoid receptor activation [62,63]. In addition, hypoxia has been shown to be a positive regulator of trophoblastic leptin expression [64]; therefore, hypoxia-inducible factor 1-alpha (HIF1A) gene expression positively correlates with leptin expression in pre-eclamptic placentas [65]. Thus, the hyperleptinemia observed in PE [66,67] is explained, at least in part, by hypoxia-induced placental production.

Concerning GDM, women with this disorder show higher circulating concentrations of leptin [68,69]. This adipokine plays a relevant role in glucose metabolism since it inhibits insulin action through the phosphorylation of serine residues in the insulin receptor substrate 1 (IRS1) [70] and insulin secretion in pancreatic beta cells [71]. This adipokine is also known to regulate insulin-mediated glucose metabolism in skeletal muscle and hepatic gluconeogenesis [72].

Also, myometrial dysfunction has been reported to be the main cause of the risk of C-section in obese women, leading to low frequency and strength of contractions [73].

Leptin stimulates the production of prostaglandin E2 (PGE2) by the placenta and adipose tissue [74], so that chronic hyperleptinemia may cause elevated PGE2 concentrations in late pregnancy and decreased sensitivity in maternal tissues to PGE2. The correct action of PGE2 is indispensable for the shortening and maturation of the cervix during preparation for labor and for better activation of labor through its action on the decidua and myometrium [75]. In addition, leptin inhibits myometrial spontaneous and oxytocin-induced contractions in vitro [76]. Interestingly, women with failed labor induction show higher leptin concentrations than women with successful labor induction to such a degree that leptin represents an independent failed labor induction predictor [77]. This inhibitory effect of leptin on myometrial contractions may also be associated with PPH since contraction of the myometrium is the main mechanism by which bleeding is stopped after delivery [78].

**Figure 3:**
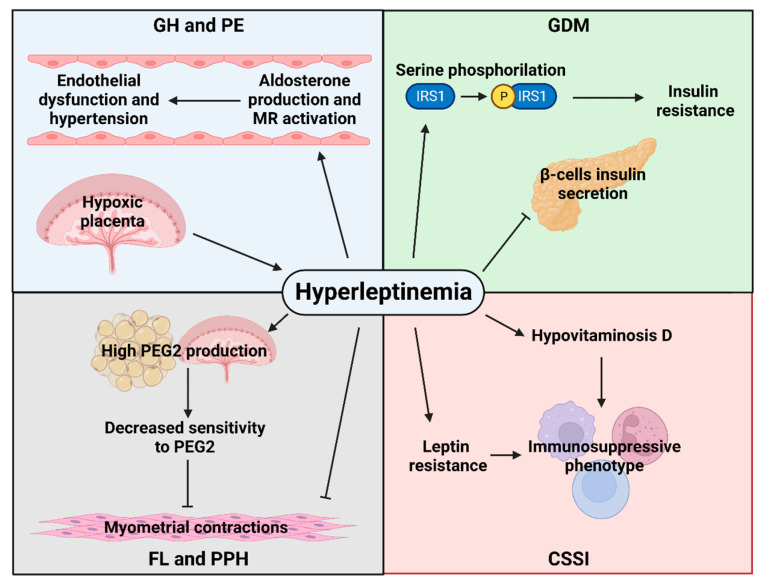
Potential pathophysiological mechanisms of hyperleptinemia in women with obesity favoring the development of maternal obstetric complications. The arrowhead lines indicate stimulation, and the T-shaped lines indicate inhibition. GH: gestational hypertension; PE: pre-eclampsia; MR: mineralocorticoid receptor; GDM: gestational diabetes mellitus; IRS1: insulin receptor substrate 1; PEG2: prostaglandin E2; FL: failed labor; PPH: postpartum hemorrhage; and CSSI: C-section surgical site infection.

For CSSI, several cohort studies have reported that obesity is a risk factor for surgical site infections [79,80,81,82]; nevertheless, the underlying mechanisms of this susceptibility have not been fully elucidated, but vitamin D deficiency and alterations in the immune system are thought to be directly involved [83]. Interestingly, a physiological interaction between vitamin D and leptin has been proposed, as their concentrations are negatively correlated [84,85]. This correlation can be explained by the inhibitory effect of vitamin D on leptin secretion by adipose tissue [86] because hypovitaminosis D contributes to leptin resistance [87], and because obesity-induced hyperleptinemia decreases the conversion of vitamin D to 25-hydroxy vitamin D [88]. Hypovitaminosis D has been reported to increase susceptibility to systemic infections [89], partly because vitamin D enhances immunity, protecting against pathogens [90]. Furthermore, although leptin activates both innate and adaptive immunity cells, the leptin resistance inherent to excessive weight may result in an immunosuppressive phenotype [83]. All these potential pathophysiological mechanisms of leptin are summarized in Figure 3.

### 5.2. Adiponectin

Adiponectin is a 30 kDa protein hormone mainly produced by white adipose tissue and, to a lesser extent, by other tissues such as the liver and placenta. Adiponectin participates in adipocyte differentiation and in the suppression of inflammation and lipotoxicity; however, it also has anti-atherogenic and anti-diabetic systemic effects [91]. Recently, it has been observed that, in the hypothalamus, adiponectin plays a role in energy homeostasis and has anti-inflammatory effects on microglia cells [92,93]. Physiologically, maternal adiponectin concentrations decrease as pregnancy progresses [94]. 

A strong association between hypoadiponectinemia and the risk of gestational hypertension and PE has been reported [95]. Experimental studies have shown that adiponectin has a cardioprotective function in vascular endothelial and smooth muscle cells [96]. This protective action lies in its ability to induce the production of nitric oxide by the activation of endothelial nitric oxide synthase (eNOS) and prevent apoptosis through the AMP-activated protein kinase (AMPK) pathway [97,98] and by suppressing inflammation through inhibiting nuclear factor kappa B (NF-κB) pathway in endothelial cells [99,100].

Women with GDM show hypoadiponectinemia [101]. Previous studies have shown that adiponectin improves glucose utilization by the AMPK pathway in C2C12 myocytes, increases glucose uptake in skeletal muscle of mice fed a high fat/sucrose diet, and augments glucose transporter type 4 (GLUT-4) translocation and glucose uptake in rat skeletal muscle cells [102,103,104]. In healthy mice, mice fed a high-fat diet, and murine models of type 1 and type 2 diabetes, the administration of adiponectin reduces plasma glucose concentrations [91]. Interestingly, administering high doses of adiponectin in mice does not cause hypoglycemic episodes, indicating that this adipokine acts probably by inhibiting gluconeogenesis or glycogenolysis. In line with this, adiponectin significantly decreases endogenous glucose production by inhibiting gene expression of the glucose 6 phosphatase and phosphoenol pyruvate carboxy kinase in the liver [105]. Furthermore, adiponectin decreases glucose output in rat hepatocytes [106].

It has been reported that adiponectin, through the AMPK pathway, potently inhibits uterine myometrial contractions in murine models [107]. This same effect has been observed in rats [108]. This makes sense with the physiological decrease in adiponectin concentrations towards the end of pregnancy; however, it does not exclude the possibility of altered adiponectin production or overexpression of its receptor in myometrium in conditions such as PPH or failed labor.

**Figure 4:**
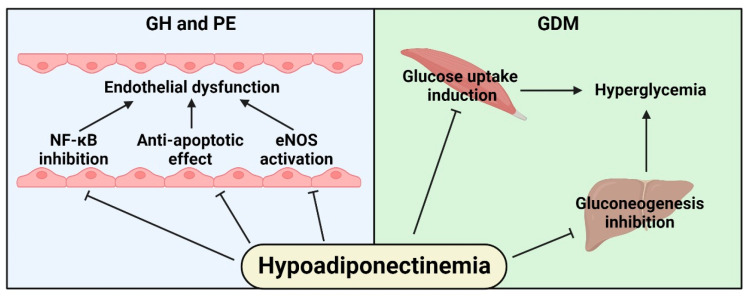
Potential pathophysiological consequences of hypoadiponectinemia in women with obesity promoting the development of maternal obstetric complications. The arrowhead lines indicate stimulation, and the T-shaped lines indicate inhibition. GH: gestational hypertension; PE: pre-eclampsia; NF-κB: nuclear factor kappa B; eNOS: endothelial nitric oxide synthase; and GDM: gestational diabetes mellitus.

All these potential pathophysiological consequences of hypoadiponectinemia are summarized in Figure 4.

## 6. Perspectives on the Study of Adipokines in Excess-Weight Pregnant Women

The following are some of the adipokines that have been studied in some obstetric complications, mainly in PE and GDM; however, to our knowledge, they have not been studied, or very little is known about them in pregnant women with excessive weight, which represents a line of research to be explored due to their key role in the regulation of glucose and lipid metabolism.

Betatrophin, a 22 kDa protein, is secreted by the liver and white adipose tissue, which plays a critical role in glucose homeostasis, lipid metabolism, and inflammation [109]. It is currently debated whether betatrophin is able to induce pancreatic β-cell proliferation and increase insulin secretion, although the consensus is that it does not have such effects [110]. Many studies have shown that betatrophin is negatively correlated with high-density lipoprotein cholesterol levels and positively correlated with triglyceride levels, suggesting that it is a key modulator of lipid metabolism [111]. The role of betatrophin in glucose and lipid homeostasis requires further investigation. Betatrophin concentrations in the third trimester of pregnancy have been reported to be higher in women with GDM than in controls [112]. The few studies of betatrophin in women with PE suggest that its levels are higher than in controls [113,114].

The pigment epithelium-derived factor (PEDF) is a 50 kDa glycoprotein secreted by the human retinal pigment epithelial cells and visceral adipose tissue. This protein induces apoptosis in macrophages, suggesting a role as an inflammation modulator. In endothelial cells, it also inhibits proliferation and migration induced by VEGF, highlighting its probable involvement in endothelial dysfunction [115]. Furthermore, PEDF induces insulin resistance in human adipocytes and skeletal muscle cells [116,117]. Recently, it has been observed that women who develop PE have high PEDF concentrations before gestational week 20 and that the PEDF/VEGF ratio has a significant negative predictive value for PE throughout pregnancy [118]. In extravillous trophoblast cells, hypoxia induces overexpression of PEDF, which is associated with increased apoptosis and decreased invasiveness that may be implicated in placental defects in PE [119]. It has been observed that PEDF concentrations are higher in women with GDM than in controls and significantly predict the development of DM2 at 3 months postpartum [120]; however, another study reports no difference in PEDF concentrations between women with GDM and controls [121].

Neutrophil gelatinase-associated lipocalin (NGAL) or lipocalin-2 (LCN-2) is a 25 kDa glycoprotein secreted by visceral adipose tissue and kidney, liver, and other tissues [122]. This glycoprotein participates in the regulation of immune response, inflammation [123], and glucose metabolism, but with regard to the latter, different studies have reported discrepant results, so it is not clear whether it promotes glucose intolerance and insulin resistance or whether it improves insulin sensitivity and glucose metabolism [124]. In women with GDM, maternal and cord blood NGAL concentrations and their placental expression are higher than in controls. Interestingly, NGAL maternal concentrations positively correlated with various parameters of insulin resistance [122]. Elevated concentrations of this glycoprotein in the first trimester of gestation are significantly associated with the risk of developing GDM [124]. Women with PE show higher NGAL concentrations than controls throughout pregnancy [125].

Fetuin-A is a multifunctional glycoprotein mainly secreted by the liver but also produced in the adipose tissue. This adipokine has been implicated in insulin resistance and metabolic syndrome because it is a natural inhibitor of insulin receptor tyrosine kinase and promotes lipid-induced insulin resistance through the activation of Toll-like receptor 4 [126,127,128]. In pregnant women, fetuin-A concentrations increase as pregnancy progresses, but the increase is significantly greater in women who develop GDM [129]. Fetuin-B is the second member of the fetuin family. Compared to fetuin-A, fetuin-B shows significant differences at the primary sequence level but is also implicated in impaired insulin action as well as glucose intolerance. GDM is characterized by an increase in the concentration of fetuin-B as compared to a normoglycemic pregnancy [130].

Progranulin is an 88 kDa protein secreted by various cell types, such as endothelial cells, fibroblasts, adipocytes, and neurons [131]. In the nervous system, it plays a key role in the development and maintenance of neurons and microglia [132]. In addition, it has been observed that this protein has anti-inflammatory properties in arthritis and participates in insulin resistance in models of obesity induced by a high-fat diet [133,134]. Interestingly, in pregnant women with obesity, progranulin concentrations positively correlate with gestational weight gain. Incubation of human uterine smooth muscle cells in serum from obese pregnant women alters the expression of contraction-associated protein genes (PTGFR, OXTR, and GJA1). It was observed that stimulation of these cells with progranulin resulted in dose-dependent suppression of GJA1 mRNA levels [135]. Women with PE have higher serum concentrations and trophoblast protein expression of progranulin than controls [136,137]. In women with GDM, progranulin concentrations are similar to those of women with normal glucose tolerance [138].

Nesfatin-1 is an 82 amino acid protein secreted by the hypothalamus and peripheral tissues such as adipose, pancreas, and duodenum tissues. One of the major functions of this adipokine is the regulation of glucose metabolism. Nesfatin-1 improves insulin sensitivity and increases glucose uptake in peripheral tissues through the Akt/AMPK/TORC2 pathway and can suppress hepatic gluconeogenesis by inhibiting the mTOR-STAT3 pathway [139,140]. In addition, higher levels of nesfatin-1 enhance glucose-induced insulin secretion by stimulating Ca^2+^ entry through L-type channels [141]. Women with GDM have similar nesfatin-1 concentrations as controls [142], but women with PE have lower concentrations of this adipokine [143,144].

Omentin-1 is an adipokine with a molecular weight of approximately 35 kDa, mainly secreted by the vascular stromal fraction of the adipose tissue and, to a lesser extent, by the intestine and placenta. This adipokine stimulates glucose uptake via AMP-activated protein kinase/Akt in cultured adipocytes [145]. It also has anti-inflammatory properties, suppressing TNF-α-induced inflammation in endothelial cells and reducing TNF-α-induced monocyte adhesion in smooth muscle cells [146,147]. Women with GDM or PE have lower concentrations of omentin-1 than healthy pregnant women [148,149].

Chemerin is a protein with several biologically active isoforms that vary in the number of amino acids (from 155 to 158 residues). This adipokine regulates angiogenesis, adipogenesis, and energy metabolism and is secreted by various tissues, particularly white adipose tissue and the liver. Since the circulating concentrations of chemerin are positively correlated with insulin resistance, BMI, and serum triglycerides, it has been suggested that chemerin may play an important role in metabolic diseases; however, this is not yet conclusive [150]. Women with GDM or PE have higher chemerin levels than healthy pregnant women [151,152,153].

## 7. Conclusions

Overweight and obesity in pregnancy favor the development of gestational hypertension, PE, GDM, and requiring a C-section to terminate the pregnancy; however, the risk is higher in women with obesity. In maternal obesity, the deregulation of circulating adipokines is more pronounced, mainly consisting of hyperleptinemia and hypoadiponectinemia, which may partly explain the predisposition to obstetric complications.

## Figures and Tables

**Table 1:** Adipokine concentrations throughout pregnancy in women with excessive pregestational weight.

Authors	Groups Compared	Gestational Age at Measurement	Concentrations in Women with Excessive Weight	Comments
**Leptin**				
Mazurek and Bronkowska, 2020 [28]	Overweight (*n* = 36) vs. normal weight women (*n* = 100)	After 39 weeks	↑	Leptin concentrations were directly proportional to the pregestational BMI and gestational weight gain.
Solis-Paredes et al., 2016 [29]	Overweight (*n* = 25) vs. normal weight women (*n* = 20)	37–40 weeks	↔	~
Franco-Sena et al., 2015 [30]	Overweight (*n* = 64) vs. normal weight women (*n* = 135)	5–13 weeks 20–26 weeks 30–36 weeks	↑ ↔ ↔	In overweight or obese women, leptin concentrations tended to be higher than in women of normal weight, but a significant difference was only observed in the first trimester of pregnancy.
Haghiac et al., 2013 [31]	Overweight (*n* = 37) vs. normal weight women (*n* = 32)	38.5–40 weeks	↑	Leptin concentrations increased according to the degree of excess body weight.
Ozias et al., 2015 [32]	Overweight/Obese (*n* = 21) vs. normal weight women (*n* = 17)	35–39 weeks	↑	Leptin concentration correlated positively with BMI and total body fat mass in all women.
Straughen et al., 2013 [33]	Overweight/Obese (*n* = 143) vs. normal weight women (*n* = 143)	6–10 weeks 10–14 weeks 16–20 weeks 22–26 weeks 32–36 weeks	↑ ↑ ↑ ↑ ↑	~
Savard et al., 2022 [34]	Obese (*n* = 15) vs. non obese women (BMI < 25 kg/m^2^, *n* = 45)	9.9–14.7 weeks 20.7–25.3 weeks 31.6–34.6 weeks	↑	Only one arrow is shown because mixed models for repeated measurements were used to analyze leptin concentrations considering gestational trimester and BMI category. Pregestational BMI correlated positively with leptin concentrations.
Mazurek and Bronkowska, 2020 [28]	Obese (*n* = 14) vs. normal weight women (*n* = 100)	After 39 weeks	↑	Leptin concentrations were directly proportional to the pregestational BMI and gestational weight gain.
Andersson-Hall et al., 2019 [35]	Obese (*n* = 19) vs. normal weight women (*n* = 30)	8–12 weeks 24–26 weeks 35–37 weeks	↑ ↑ ↑	The concentration of the soluble leptin receptor was also measured, and the free leptin index was calculated, which was significantly higher in obese women.In all women, changes in the free leptin index correlated positively with changes in fat mass across the pregnancy.
Hinkle et al., 2019 [36]	Obese (*n* = 66) vs. non obese women (BMI < 30 kg/m^2^, *n* = 255)	10–14 weeks 15–26 weeks 23–31 weeks 33–39 weeks	↑ ↑ ↑ ↑	The concentration of the soluble leptin receptor was also measured, and the free leptin index was calculated, which was significantly higher in obese women.
Solis-Paredes et al., 2016 [29]	Obese (*n* = 22) vs. normal weight women (*n* = 20)	37–40 weeks	↔	~
Vernini et al., 2016 [37]	Obese (*n* = 31) vs. normal weight women (*n* = 23)	Weeks 37–38	↑	~
Franco-Sena et al., 2015 [30]	Obese (*n* = 33) vs. normal weight women (*n* = 135)	5–13 weeks 20–26 weeks 30–36 weeks	↑ ↔ ↔	In overweight or obese women, leptin concentrations tended to be higher than in women of normal weight, but a significant difference was only observed in the first trimester of pregnancy.
Priyadarshini et al., 2014 [38]	Obese (*n* = 10) vs. normal weight women (*n* = 10)	36–38 weeks	↑	~
Haghiac et al., 2013 [31]	Women with BMI 30–40 (*n* = 36) vs. normal weight women (*n* = 32)	38.5–40 weeks	↑	Leptin concentrations increased according to the degree of excess body weight.
Haghiac et al., 2013 [31]	Women with BMI > 40 (*n* = 28) vs. normal weight women (*n* = 32)	38.5–40 weeks	↑	See the previous comment for this same reference.
Sen et al., 2013 [39]	Obese (*n* = 15) vs. normal weight women (*n* = 15)	24–28 weeks	↑	~
**Adiponectin**				
Savard et al., 2022 [34]	Overweight (*n* = 19) vs. non obese women (BMI < 25 kg/m^2^, *n* = 45)	9.9–14.7 weeks 20.7–25.3 weeks 31.6–34.6 weeks	↓	Only one arrow is shown because mixed models for repeated measurements were used to analyze adiponectin concentrations considering gestational trimester and BMI category. Pregestational BMI correlated negatively with adiponectin concentrations in the first and second trimesters.
Solis-Paredes et al., 2016 [29]	Overweight (*n* = 25) vs. normal weight women (*n* = 20)	37–40 weeks	↔	~
Savard et al., 2022 [34]	Obese (*n* = 15) vs. non obese women (BMI < 25 kg/m^2^, *n* = 45)	9.9–14.7 weeks 20.7–25.3 weeks 31.6–34.6 weeks	↓	See the previous comment for this same reference.
Andersson-Hall et al., 2019 [35]	Obese (*n* = 19) vs. normal weight women (*n* = 30)	8–12 weeks 24–26 weeks 35–37 weeks	↓ ↓ ↓	~
Hinkle et al., 2019 [36]	Obese (*n* = 66) vs. non obese women (BMI < 30 kg/m^2^, *n* = 255)	10–14 weeks 15–26 weeks 23–31 weeks 33–39 weeks	↓ ↓ ↓ ↓	Arrows indicate total and high molecular weight adiponectin concentrations, although the difference was greater in the latter at weeks 23–31 and 33–39.
Priyadarshini et al., 2014 [38]	Obese (*n* = 10) vs. normal weight women (*n* = 10)	36–38 weeks	↔	~
Vernini et al., 2016 [37]	Obese (*n* = 31) vs. normal weight women (*n* = 23)	37–38 weeks	↔	~
Solis-Paredes et al., 2016 [29]	Obese (*n* = 22) vs. normal weight women (*n* = 20)	37–40 weeks	↔	~
Ozias et al., 2015 [32]	Overweight/Obese (*n* = 21) vs. normal weight women (*n* = 17)	35–39 weeks	↔	Total and high molecular weight adiponectin concentrations were measured, and none were different between groups.
Haghiac et al., 2013 [31]	Women with BMI > 40 (*n* = 28) vs. normal weight women (*n* = 32)	38.5–40 weeks	↓	Adiponectin concentrations of women with BMI 25–30 and 30–40 were also compared with women with normal weight, but no significant difference was observed.
Sen et al., 2013 [39]	Obese (*n* = 15) vs. normal weight women (*n* = 15)	24–28 weeks	↓	~
**Resistin**				
Solis-Paredes et al., 2016 [29]	Overweight (*n* = 25) vs. normal weight women (*n* = 20)	37–40 weeks	↔	~
Vernini et al., 2016 [37]	Obese (*n* = 31) vs. normal weight women (*n* = 23)	37–38 weeks	↔	~
Ozias et al., 2015 [32]	Overweight/Obese (*n* = 21) vs. normal weight women (*n* = 17)	35–39 weeks	↔	In all women, resistin concentration correlated positively with abdominal visceral fat mass relative to total body fat mass.
Solis-Paredes et al., 2016 [29]	Obese (*n* = 22) vs. normal weight women (*n* = 20)	37–40 weeks	↔	~
**TNF-α**				
Maguire et al., 2021 [40]	Obese (*n* = 124) vs. non obese women (BMI < 30 kg/m^2^, *n* = 237)	Median = 11.6 weeks (IQR = 9.3–18.7)	↑	Maternal concentrations of IL-8, IL-1β, IL-4, IFN-γ, IL-12 p70 subunit, and IL-17A were also analyzed, but no significant differences were observed.
**IL-6**				
Savard et al., 2022 [34]	Overweight (*n* = 19) and obese (*n* = 15) vs. non obese women (BMI < 25 kg/m^2^, *n* = 45)	9.9–14.7 weeks 20.7–25.3 weeks 31.6–34.6 weeks	↔	Only one arrow is shown because mixed models for repeated measurements were used to analyze IL-6 concentrations considering gestational trimester and BMI category. Pregestational BMI correlated positively with IL-6 concentrations in the first and second trimesters.
Maguire et al., 2021 [40]	Obese (*n* = 124) vs. non obese women (BMI < 30 kg/m^2^, *n* = 237)	Median = 11.6 weeks (IQR = 9.3–18.7)	↔	~
**Visfatin**				
Ozias et al., 2015 [32]	Overweight/Obese (*n* = 21) vs. normal weight women (*n* = 17)	35–39 weeks	↔	~
**Apelin**				
Hanssens et al., 2022 [41]	Obese (*n* = 30) vs. normal weight women (*n* = 36)	35–40 weeks	↔	~

Weight status was determined based on pregestational weight. BMI: body mass index; TNF-α: tumor necrosis factor-alpha; IL-6: interleukin 6; IQR: interquartile range. ↑ indicates statistically significant higher concentrations in women with excessive weight than controls; ↓ indicates statistically significant lower concentrations in women with excessive weight than controls; ↔ indicates no statistical difference in concentrations between groups; ~ indicates that there is no association between the adipokine and pre-pregnancy BMI or that the study has no feature to highlight.

## Data Availability

No new data were created or analyzed in this study. Data sharing is not applicable to this article.

## Supplementary Materials

The following supporting information can be downloaded at https://www.mdpi.com/article/10.3390/ijms241914678/s1.
