# Supplementary material for: Excessive Pregestational Weight and Maternal Obstetric Complications: The Role of Adipokines
Source: Int J Mol Sci. 2023 Sep 28;24(19):14678. doi: 10.3390/ijms241914678 (PMC10572963; doi:10.3390/ijms241914678)
Supplement: Supplementary file 1 [file ijms-24-14678-s001.zip › ijms-2625020-supplementary.pdf]

Table S1. Prevalence of excessive weight in women from different countries. There are no data recorded for Chile, Colombia, and Costa Rica.

Country	Period	Age range	Obesity	Overweight	Overweight + Obesity
Mexico	2018-2019	20-39	32.6%	36.6%	69.2%
United States	2017-2018	18-44	39.6%	25.1%	64.7%
New Zealand	2019-2020	15-44	27.3%	27.1%	54.4%
Slovenia	2019	18-44	28.0%	21.2%	49.2%
Canada	2008	20-44	19.8%	25.8%	45.6%
Australia	2014-2015	18-44	21.8%	23.2%	45.0%
Ireland	2021-2022	15-44	17.3%	25.0%	42.3%
Türkiye	2019	18-44	14.0%	27.6%	41.6%
Finland	2019	18-44	15.2%	26.3%	41.5%
Iceland	2014	18-44	15.7%	22.6%	38.3%
Netherlands	2020	20-40	11.4%	25.3%	36.7%
United Kingdom	2017	16-34	16.1%	20.5%	36.6%
Hungary	2019	18-44	13.4%	20.5%	33.9%
Norway	2019	18-44	10.6%	23.2%	33.8%
Germany	2019	18-44	12.2%	21.3%	33.5%
Portugal	2019	18-44	11.6%	21.5%	33.1%
Denmark	2019	18-44	13.6%	19.3%	32.9%
Belgium	2019	18-44	10.0%	22.8%	32.8%
Israel	2014-2016	18-44	12.0%	20.5%	32.5%
Sweden	2020	18-44	11.5%	21.0%	32.5%
France	2019	18-44	11.3%	20.0%	31.3%
Spain	2019	18-44	9.2%	21.0%	30.2%
Czech Republic	2019	18-44	9.6%	20.5%	30.1%
Austria	2019	18-44	9.6%	20.4%	30.0%
Latvia	2019	18-44	9.0%	20.3%	29.3%
Slovak Republic	2019	18-44	7.8%	21.4%	29.2%
Poland	2019	18-44	8.7%	20.3%	29.0%
Estonia	2019	18-44	10.7%	17.3%	28.0%
Lithuania	2019	18-44	9.1%	18.5%	27.6%
Luxembourg	2019	18-44	9.4%	17.8%	27.2%
Greece	2019	18-44	7.3%	18.7%	26.0%
Italy	2019	18-44	5.1%	16.4%	21.5%
Switzerland	2012. The most recent data are from 2014-2015 but are not separated by gender.	15-44	5.3%	14.5%	19.8%
